# Structure of the metazoan Rab7 GEF complex Mon1–Ccz1–Bulli

**DOI:** 10.1073/pnas.2301908120

**Published:** 2023-05-08

**Authors:** Eric Herrmann, Jan-Hannes Schäfer, Stephan Wilmes, Christian Ungermann, Arne Moeller, Daniel Kümmel

**Affiliations:** ^a^Department of Chemistry and Pharmacy, Institute of Biochemistry, University of Münster, 48149 Münster, Germany; ^b^Department of Biology/Chemistry, Structural Biology section, Osnabrück University, 49076 Osnabrück, Germany; ^c^Department of Biology/Chemistry, Biochemistry section, Osnabrück University, 49076 Osnabrück, Germany; ^d^Center of Cellular Nanoanalytic Osnabrück, Osnabrück University, 49076 Osnabrück, Germany

**Keywords:** cryo-EM, GEF, GTPase, membrane trafficking, endosomal maturation

## Abstract

The endosomal system of eukaryotic cells represents a central sorting and recycling compartment linked to metabolic signaling and the regulation of cell growth. Tightly controlled activation of Rab GTPases is required to establish the different domains of endosomes and lysosomes. In metazoans, Rab7 controls endosomal maturation, autophagy, and lysosomal function. It is activated by the guanine nucleotide exchange factor (GEF) complex Mon1–Ccz1–Bulli (MCBulli) of the tri-longin domain (TLD) family. While the Mon1 and Ccz1 subunits have been shown to constitute the active site of the complex, the role of Bulli remains elusive. We here present the cryo-electron microscopy (cryo-EM) structure of MCBulli at 3.2 Å resolution. Bulli associates as a leg-like extension at the periphery of the Mon1 and Ccz1 heterodimers, consistent with earlier reports that Bulli does not impact the activity of the complex or the interactions with recruiter and substrate GTPases. While MCBulli shows structural homology to the related ciliogenesis and planar cell polarity effector (Fuzzy–Inturned–Wdpcp) complex, the interaction of the TLD core subunits Mon1-Ccz1 and Fuzzy–Inturned with Bulli and Wdpcp, respectively, is remarkably different. The variations in the overall architecture suggest divergent functions of the Bulli and Wdpcp subunits. Based on our structural analysis, Bulli likely serves as a recruitment platform for additional regulators of endolysosomal trafficking to sites of Rab7 activation.

## Significance

Tri-longin domain (TLD) RabGEFs represent a group of protein complexes that play crucial roles in the organization of endosomes, lysosomes, and lysosome-related organelles. During evolution, the TLD family has diversified from one dimeric complex in yeast to three complexes with up to four subunits in metazoans. How this expansion contributes to different functions and regulation remains elusive. Our cryo-electron microscopy (cryo-EM) structure of the trimeric Mon1–Ccz1–Bulli TLD RabGEF from fruit flies provides a framework to address these questions and to understand the implications for the organization of the endolysosomal system.

Rab GTPases play essential roles in intracellular trafficking and maintenance of organelle identity (1). They are converted from a GDP (guanosine diphosphate) “off” state into a GTP (guanosine triphosphate) “on” state by guanine nucleotide exchange factors (GEFs) and inactivated by GTPase activating proteins (GAPs). Only active GTP-bound Rabs bind effector proteins that mediate downstream functions. In the spatiotemporal control of Rab activity, the cognate RabGEFs assume a vital role as the most upstream regulators.

Different RabGEFs display a remarkable diversity in their structures and catalytic mechanisms. In addition to the Vps9 domain (2), DENN (differentially expressed in normal and neoplastic cells) domain (3, 4), and coiled-coil-containing proteins (5–7), the multisubunit “transport protein particle” (TRAPP) (8, 9) and tri-longin domain (TLD) (10, 11) complexes have been characterized as RabGEFs. Interestingly, different TRAPP isocomplexes that vary in subunit composition were described (12–15). Depending on the species or the cellular context, particular subunits can alter the substrate specificity and contribute to TRAPP localization. One representative of the TLD RabGEFs is present in the fungal genome, whereas three different complexes exist in metazoans (16, 17). The “biogenesis of lysosome-related organelles complex-3” (BLOC-3) and “ciliogenesis and planar cell polarity effector” (CPLANE) complex, named “planar cell polarity effector” (PPE) in *Drosophila melanogaster*, are only found in metazoans. BLOC-3 comprises the TLD proteins Hps1 and Hps4 and activates Rab32 and Rab38 to form lysosome-related organelles (18). Mutations in the *HPS1* and *HPS4* genes cause the name-giving disease Hermansky–Pudlak syndrome (19). CPLANE contains the TLD core subunits Fuzzy and Inturned that additionally bind the subunit Wdpcp and the noncanonical GTPase Rsg1 (20). A catalytic glutamine residue and a lipidation motif, usually hallmarks of small GTPases, are missing in Rsg1. Fuzzy and Inturned form together the cognate GEF for Rab23 and function within the CPLANE and PPE complexes in Hedgehog signaling, ciliogenesis, and establishing planar cell polarity (17, 21).

**Fig. 1.**
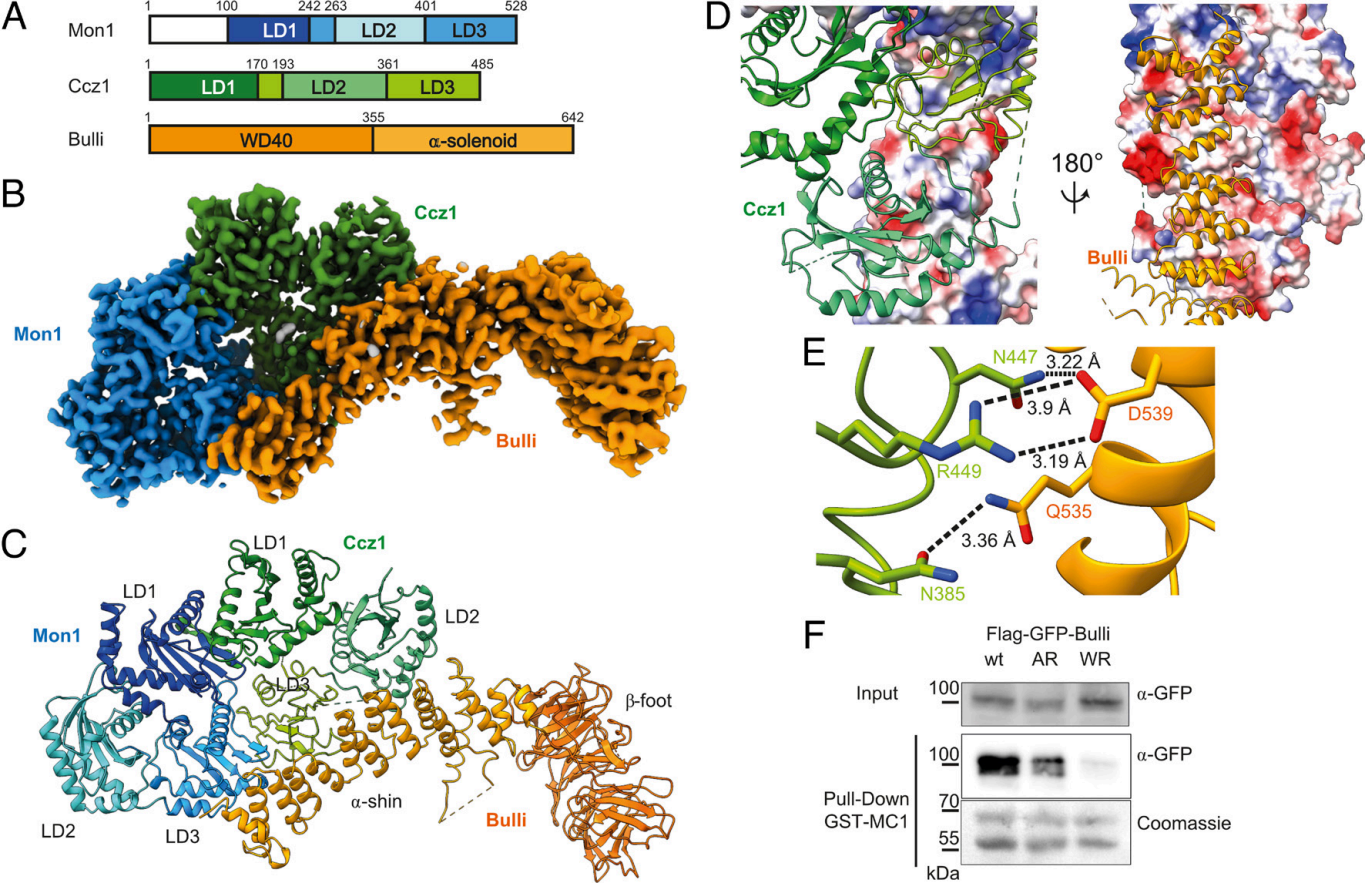
Structure of MCBulli. (*A*) Domain organization and boundaries of the *Drosophila melanogaster* subunits. (*B*) 3D reconstruction of the Mon1–Ccz1–Bulli complex at 3.2 Å resolution. (*C*) Overall architecture of the Mon1 (blue)–Ccz1 (green)–Bulli (orange) with labels of the individual subunit domains. (*D*) Interaction interface of Ccz1–LD2/3 with the Bulli α-solenoid. (*E*) Key polar interactions between conserved residues of Ccz1 and Bulli. (*F*) Pull-down assay with GST-Mon1 and 3×Flag-Ccz1 immobilized on GSH agarose and incubated with 3×Flag-GFP-Bulli expressed in HEK293 cells. Samples were analyzed by SDS-PAGE and western blotting as indicated. Representative of three independent experiments is shown.

The sole TLD RabGEF in yeast is the dimeric Mon–Ccz1 (MC1) complex, which activates Rab7, an essential organizer of the late endosomal and lysosomal compartments in eukaryotic cells (11, 17). The structure of *Chaetomium thermophilum* MC1 showed that the LD of each subunit are arranged in a triangular fashion and assembled into a pseudo-symmetric dimer (22). MC1 forms two layers: the first LDs of both subunits form the catalytic site on top, which binds to and stabilizes the nucleotide-free form of Ypt7 and thus promotes nucleotide exchange (10). The remaining LDs assemble as a base that mediates membrane interactions via electrostatic interactions and an amphipathic helix (23). The Mon1–Ccz1 TLD subunits in metazoans bind an additional protein named RMC1 in humans and Bulli in flies (Fig. 1*A*) (24–26). The intact trimeric complex is required for proper Rab7 function in endosomal maturation, autophagy, and lysosomal cholesterol transport. However, Bulli does not influence the activity of MC1 toward Rab7 in vitro (24, 27).

To gain insight into the function of Bulli, we determined the cryo-electron microscopy (cryo-EM) structure of the Mon1–Ccz1–Bulli (MCBulli) complex. The analysis of the structure reveals that Bulli does not influence the core functions of the TLD core complex but likely represents a docking platform for additional regulators of endosomal trafficking. Our structure thus provides a framework to understand the complex mechanisms of Rab regulation in metazoan cells.

## Results

We determined the cryo-EM structure of the full-length MCBulli complex, only lacking the first 100 residues of Mon1 that were removed to increase complex stability. The final consensus map had a resolution of 3.2 Å and allowed us to build a high-confidence model of the complex (Fig. 1 *B* and *C* and *SI Appendix*, Figs. S1–S3 and Table S1). The structure of the Mon1–Ccz1 TLD dimer subcomplex of *Drosophila melanogaster* (*Dm*) strongly resembles the structure of the MC1 complex from the fungus *Chaetomium thermophilum* (*Ct*), with high structural similarity of the LD1/LD1 core domains that bind the substrate GTPase Rab7 or Ypt7, respectively (rmsd of 1.96 Å over 183 residues). As expected, the catalytic site at the LD1/LD1 top layer also shows a high degree of conservation of the surface residues (*SI Appendix*, Fig. S4*D*). We also note a second conserved site on the surface of Mon1 at LD2 that does not belong to the active site.

Bulli adopts a leg-like structure composed of an N-terminal β-propeller “foot” and a C-terminal α-solenoid “shin” (Fig. 1*C*). Binding to Mon1–Ccz1 is mediated by the α-shin domain, mainly via interactions with LD2 and LD3 of Ccz1 and a few contacts with LD3 of Mon1. The β-foot reaches away from Mon1–Ccz1 and shows some flexibility, describing an approximately 5° bending motion according to variability analysis of 3D intermediate reconstructions (*SI Appendix*, Fig. S2*B*). The extensive interface between Bulli and Mon1-Ccz1 (2,021 Å^2^) is dominated by polar interactions (Fig. 1*D*), including a conserved salt bridge between Bulli^D539^–Ccz1^R449^ and hydrogen bonds between Bulli^D539^–Ccz1^N447^ and Bulli^Q535^–Ccz1^N385^ (Fig. 1*E*). These positions are conserved, pointing at a key role of these contacts in the assembly of the trimeric complex. To test the significance of the residues for the formation of the interface, we generated two Bulli mutants Q535A/D539R (AR) and Q535W/D539R (WR). In pull-down assays, the ability of these variants to bind MC1 was drastically reduced compared to wild-type Bulli (Fig. 1*F*). As expected, the effect was more pronounced for the WR variant, which disrupts the interface with a sterically demanding hydrophobic residue in addition to a charge inversion. These results confirm the relevance of the conserved polar residues for mediating formation of the MCBulli complex.

**Fig. 2.**
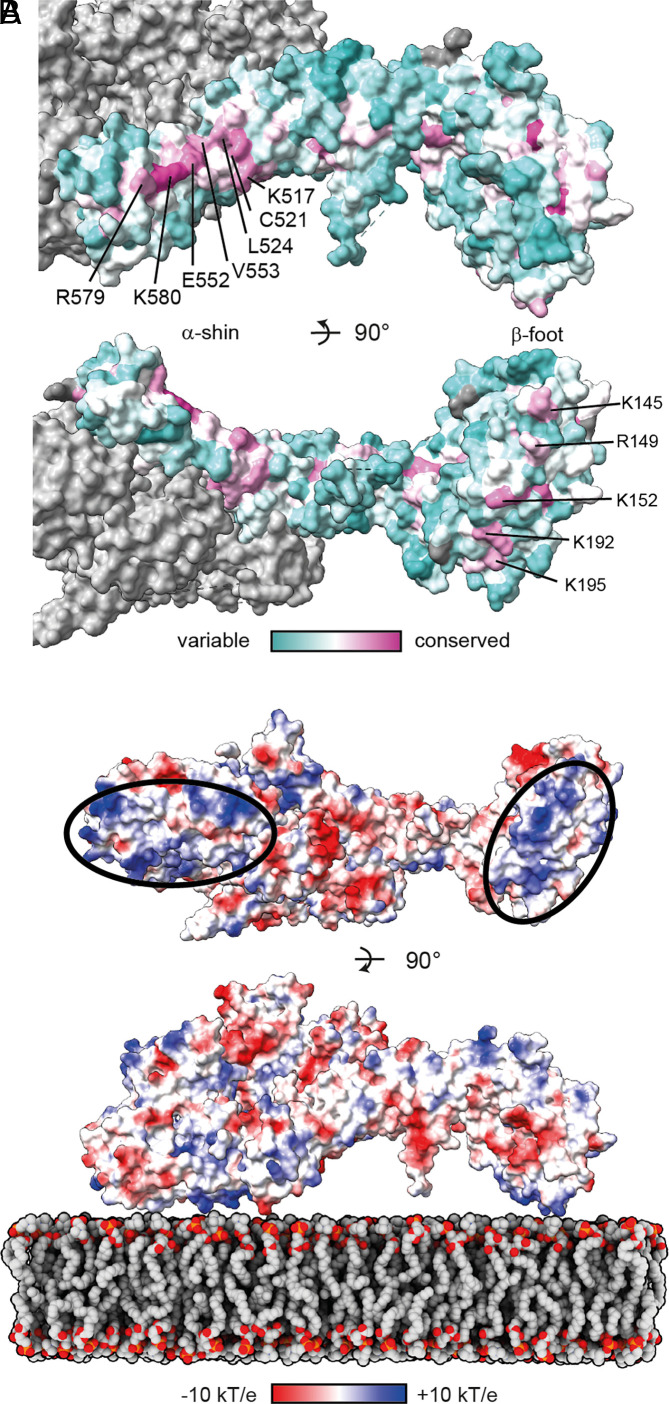
Surface properties of MCBulli. (*A*) Conserved surface patches at the α-shin and β-foot of Bulli. (*B*) Basic patches on Mon1 LD2 and the Bulli β-foot align in one plane that could represent the membrane interaction interface.

Interestingly, the surface of Bulli contains an extensive conserved surface patch (28), running alongside the α-shin that points away from Mon1–Ccz1 (Fig. 2*A* and *SI Appendix*, Fig. S4*D*). It is tempting to speculate that this region may represent a binding site for factors that contribute to MCBulli function. An additional conserved site on Bulli is found at LD2 of Mon1 and at the tip of the Bulli β-foot (Fig. 2*A*).

We previously showed that yeast MC1 contains a cluster of basic residues at LD2 and LD3 of Mon1 that positions the complex on lipid bilayers (22, 23). A similar basic patch is observed on *Dm* MCBulli, suggesting that membrane interaction may occur similarly (Fig. 2*B* and *SI Appendix*, Fig. S4*C*). In this orientation, a basic region at the β-foot that corresponds to the conserved patch (Fig. 2*A*) will also face toward the membrane. From these observations, we hypothesize that MCBulli attaches to the anionic membrane surface via two basic regions at opposing ends of the complex, thus orienting the complex on the organelle.

**Fig. 3.**
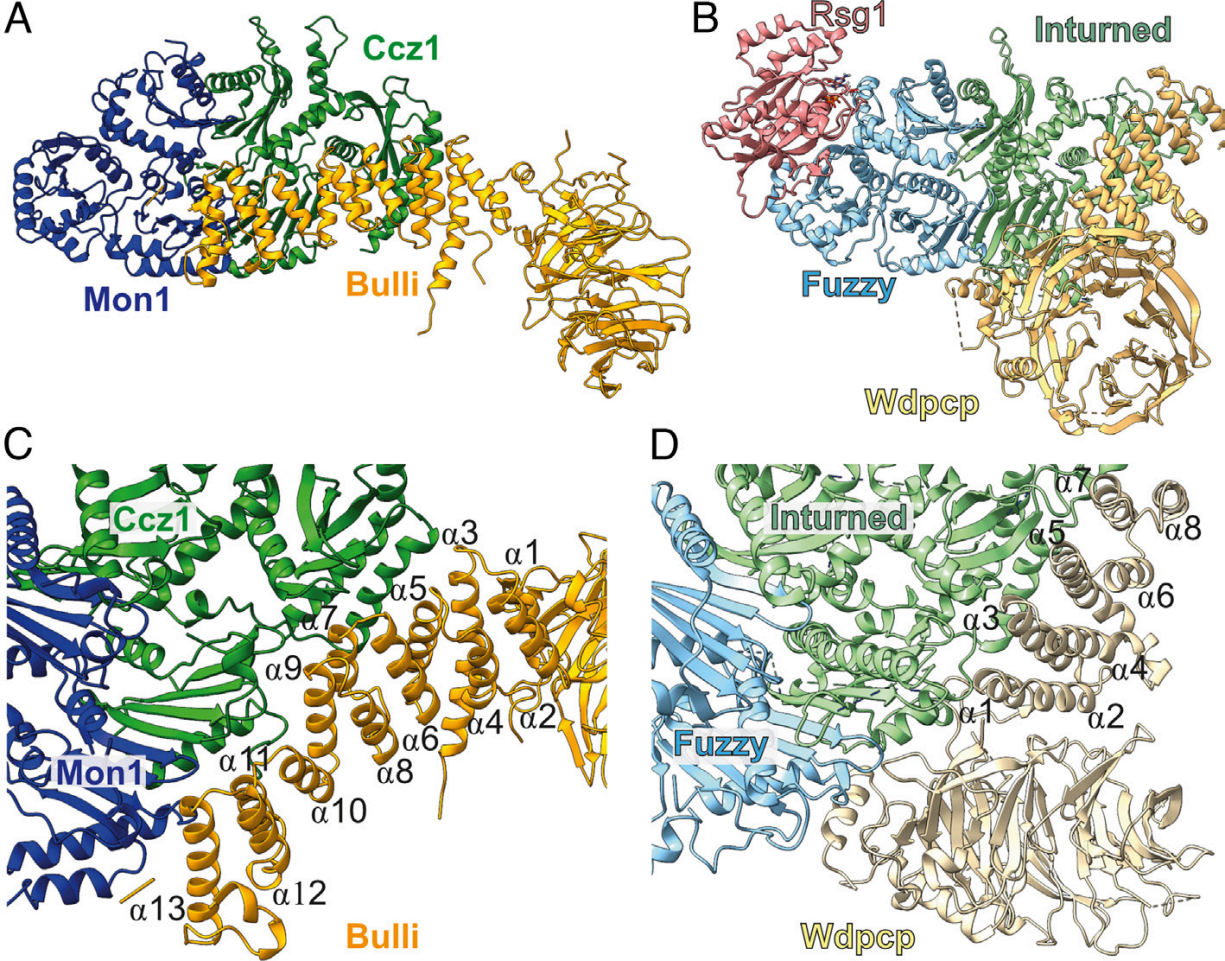
Structural divergence and conservation of TLD RabGEFs. (*A*) Structure of the MCBulli and (*B*) CPLANE complexes shown in the same orientation of the TLD subunits. (*C*) Interaction of Bulli (orange) with the TLD Mon1 (blue)/Ccz1 (green) heterodimer. (*D*) Interaction of Wdpcp (yellow) with the TLD Fuzzy (cyan)/Inturned (light green) heterodimer. The helices of the α-solenoid domains of Bulli and Wdpcp (labeled α1-α12 and α1-α8, respectively) run alongside of Ccz1 and Inturned in opposite directions.

The structure of MCBulli resembles the CPLANE complex (29), which is composed of the homologous subunits Fuzzy (Mon1), Inturned (Ccz1), Wdpcp (Bulli), and additionally the noncanonical GTPase Rsg1 (Fig. 3 *A* and *B*). The structures of the Mon1–Ccz1 and Fuzzy–Inturned heterodimers share a similar overall architecture (rmsd of 3.6 Å over 663 residues). The β-propellers of Bulli and Wdpcp both have an unusual fold, which involves a Velcro closure by an N-terminal extension. This element provides the final β-strand of the seventh blade and a parallel strand interaction with an insertion after blade six. However, the α-solenoid is much longer in Bulli (15 instead of 8 helices in Wdpcp). Although the interaction sites of Wdpcp and Bulli are on the same face of the TLD core, there are remarkable differences (Fig. 3 *C* and *D*). Both interfaces are polar, but Ccz1–Bulli does not show the prominent complementary charge distribution like Inturned–Wdpcp. The orientations of Bulli and Wdpcp relative to the tri-longin core are flipped, and while the Bulli β-foot points away from Mon1–Ccz1, the Wdpcp propeller makes extensive contact with Fuzzy.

**Fig. 4.**
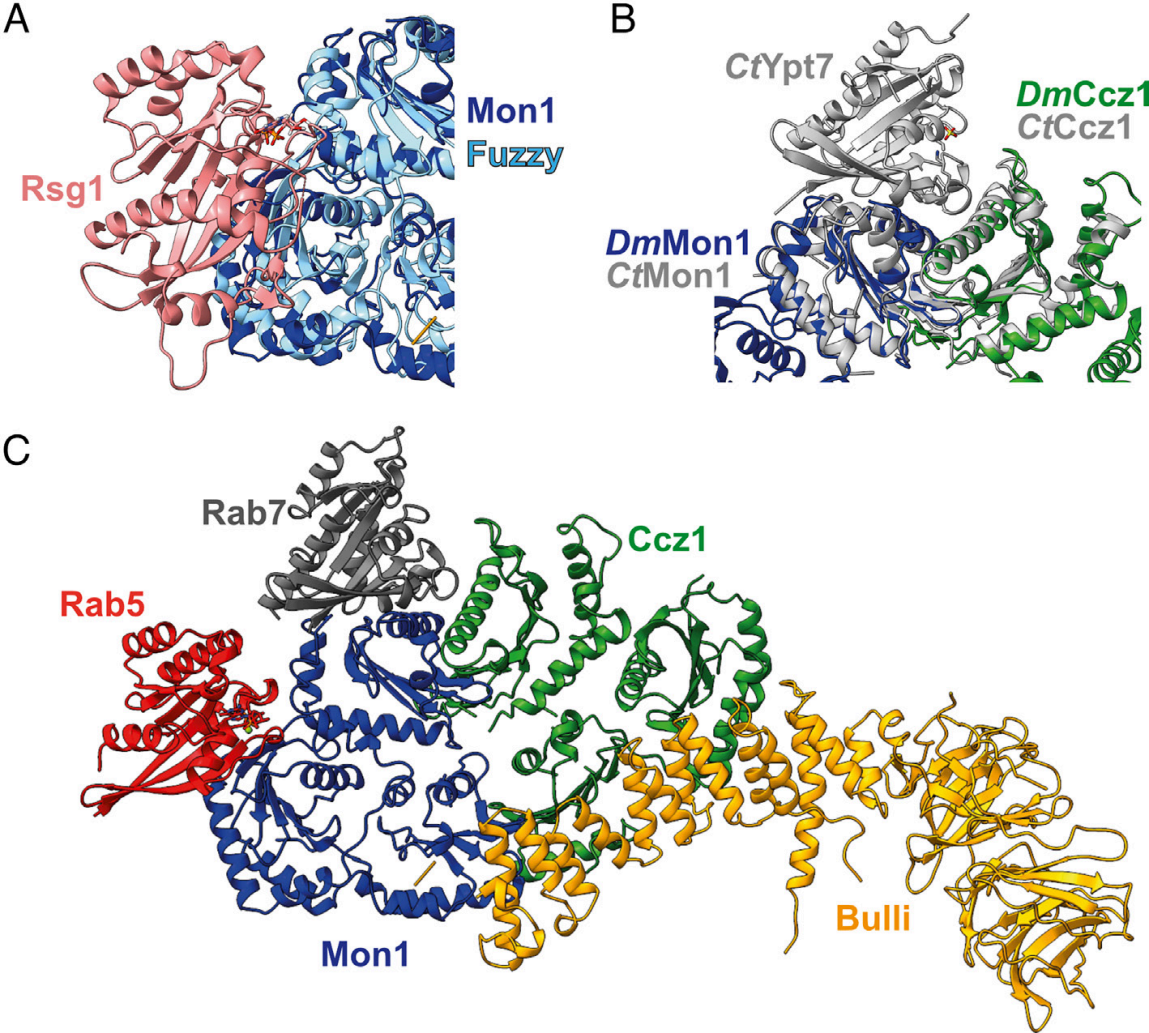
Model of MCBulli bound to recruiter and substrate GTPases. (*A*) Superposition of Rsg1 (pink)/Fuzzy (cyan) with Mon1 (blue). (*B*) Superposition of *Chaetomium thermophilum* MC1LD1core/Ypt7 complex (light gray) with *Drosophila melanogaster* MCBulli. (*C*) Composite model of the pentameric GTP Rab5 (red)/Mon1 (blue)/Ccz1 (green)/Bulli (orange)/Rab7 (dark gray) complex from the combination of experimental and modeling data (see *Method* section for details).

Rsg1 interacts with CPLANE in an effector mode at LD1 and LD2 of Fuzzy, a site that also shows a high degree of conservation on Mon1 (Fig. 3*B* and *SI Appendix*, Fig. S4*B*). Considering that MCBulli is an effector of Rab5, it is very likely that Rsg1–CPLANE and Rab5–MCBulli interact similarly (Fig. 4*A*). Indeed, the GTPase domain of Rab5 could be readily modeled onto MCBulli using AlphaFold2 (30). The catalytic LD1 core complex of fungal MC1 (10) could also be aligned well with the MCBulli structure (Fig. 4*B*), which allowed us to complement the complex with a model of nucleotide-free Rab7. The resulting experimental and prediction-based composite model (see *SI Appendix*, *Methods* for details) of the Rab5 GTP–MCBulli–Rab7 complex (Fig. 4*C*) provides a molecular framework to understand MCBulli function. Notably, Bulli binds remotely of the interaction sites of Mon1 and Ccz1 with membranes, the recruiter GTPase Rab5 or the substrate Rab7. Furthermore, no conformational changes of the TLD core between the dimeric *Ct* MC1 and trimeric *Dm* MCBulli can be observed (Fig. 4*B* and *SI Appendix*, Fig. S4*A*), excluding allosteric effects from Bulli binding. Consequently, no influence of Bulli on the activities of the Mon1–Ccz1 core subcomplex is to be expected, pointing at a role of Bulli as an adaptor that expands the interaction spectrum of the complex.

## Discussion

The conclusions from the structural analysis of MCBulli are consistent with those of previous studies that showed no difference in trimeric MCBulli function compared to dimeric MC1 in biochemical assays: Neither binding to Rab5 nor activity toward Rab7 is affected by the presence of Bulli, consistent with the binding sites for the GTPases and Bulli being far apart (24, 27). Thus, rather than acting as a regulator of Mon1–Ccz1 function, Bulli likely functions as an adaptor for additional factors to sites of Rab7 activation. An attractive candidate for such a factor is TBC1D18 (CenAGAP in flies), a Rab5 GAP that was recently described to interact indirectly with MC1 (31). We speculate that Bulli may be proposed “factor X” that links TBC1D18 to MC1, possibly via the conserved interface on the α-shin (Fig. 2*B*). This would allow concurrent removal of Rab5 from sites of Rab7 activation and thus may explain defective Rab5 inactivation observed in Bulli knockout flies (24). The presence of a conserved basic patch on Bulli that aligns with the lipid-binding site on Mon1 may also contribute to the membrane association of the complex. Proper positioning of GEF complexes is a key aspect in the recognition of substrate GTPases (13, 32), which is in line with the essential role of Bulli and its homologs in a physiological setting (24–26).

The comparison of MCBulli with the CPLANE complex reveals several conserved features. The metazoan-specific subunits Bulli and Wdpcp are both β-propeller/α-solenoid proteins with a characteristic topology and bind to equivalent sites on the TLD subcomplex. Given this evolutionary relationship of Bulli and Wdpcp, TLD RabGEFs likely first acquired a third subunit and then further diversified. Bulli and Wdpcp show divergent evolution and thus may mediate the specific functions of the MCBulli and CPLANE complexes in distinct cellular pathways. Furthermore, MCBulli and CPLANE may contain a conserved binding site for a GTPase on Mon1 and Fuzzy, respectively. Functional studies on the regulation of the yeast MC1 and *Drosophila* MCBulli complexes provide experimental support for the proposed Rab5–MCBulli binding site (33). However, in contrast to Rab5, Rsg1 does not contain a lipidation motif and is unlikely a recruiter of CPLANE to membranes. With the sequence features of a GTP-locked active GTPase, Rsg1 may be a constitutive subunit of CPLANE required for GEF activity or regulation.

The striking resemblances of MCBulli and CPLANE raise the question of whether the common features of these complexes may also be conserved for the third TLD RabGEF, the BLOC-3 complex. BLOC-3 is an effector of Rab9 (34); however, Hps4 (homologous to Ccz1 and Inturned) was identified as the Rab9-interacting subunit. Also, we could not model an interaction of Rab9 with BLOC-3 using AlphaFold2 (30, 35). Thus, if Rab9 serves as a recruiter and activator for BLOC-3, the mechanism is likely different from Rab5 for MC1. Regarding an accessory β-propeller/α-solenoid subunit, BLOC-3 may have lost its third subunit again during evolution. Interestingly, the Hermansky–Pudlak syndrome gene *HPS6* encodes an β-propeller/α-solenoid protein. Hps6 was described as a subunit of the BLOC-2 complex (36) but may have a dual function in separate complexes like it was described for Sec13 (37–39). Alternatively, a so far unidentified β-propeller/α-solenoid protein may bind to Hps1-Hps4. Revisiting the BLOC-3 interactome may thus be worthwhile.

## Materials and Methods

### Protein Expression and Purification.

A complex of *Dm*Mon1-ΔN (residues 101-528, N-terminal GST-tag/PreScission cleavage site), *Dm*Ccz1-fl (residues 1 to 485, N-terminal 3×Flag-tag), and *Dm*Bulli-fl (residues 1 to 642, N-terminal 6×His-tag) was produced in *Spodoptera frugiperda* 21 cells using the biGBac expression system (40). The complex was isolated from cell lysate on a glutathione affinity matrix, followed by PreScission protease cleavage overnight. Elution fractions were further purified by Ni-NTA (nickel-nitrilotriacetic acid) affinity chromatography and size exclusion chromatography (*SI Appendix*, Fig. S1). Peak fractions were directly used for cryo-EM sample preparation.

### Cryo-EM Sample Preparation and Data Collection.

Purified MCBulli was applied to glow-discharged CF-1.2/1.3-3 Cu-50 grids and plunge frozen in liquid ethane using a Vitrobot Mark IV (ThermoFisher Scientific). Data were collected on a Glacios electron microscope equipped with a Selectris energy filter and a Falcon Mark IV direct electron detector (ThermoFisher Scientific) at 130,000-fold nominal magnification.

### Cryo-EM Data Processing, Model Building, and Refinement.

A total of 5,931 movies were collected automatically in EPU (Thermo Fisher), motion-corrected, and filtered by the contrast transfer function fit estimates using a cutoff at 5 Å in cryoSPARC live (41). A total of 4,842 micrographs were selected for data processing. Well-aligning two-dimensional class averages were subjected to iterative rounds of heterogeneous and nonuniform refinements in cryoSPARC (41) to yield a consensus reconstruction at 3.2 Å resolution. Local refinement of the MCBulli core and the beta-propeller were used to generate a composite model with a resolution ranging from 3.2 to 3.7 Å. The final composite map was sharpened with DeepEMhancer (42). Model building started from AlphaFold (30) predictions that were iteratively refined using Coot (43) and Phenix (44).

### Pull-Down Assay.

Bulli wild type and mutants expressed in HEK293T cells were incubated with purified MC1 complex immobilized on GSH beads. Reactions were washed and analyzed by sodium dodecyl sulfate–polyacrylamide gel electrophoresis (SDS-PAGE) and western blotting for Bulli binding to GST-MC1.

### Computational Modeling.

The pentameric *Drosophila* Rab5 GTP/Mon1/Ccz1/Bulli/Rab7 complex was modeled by combining the Mon1/Ccz1/Bulli structure with predictions of the dimeric Rab5/Mon1 and the trimeric Mon1/Ccz1/Rab7 complexes from AlphaFold2 multimer (30, 35) (*SI Appendix*, Fig. S5). The conformations of the GTPases were adjusted based on the structure of human Rab5A (45) (PDB ID 1TU3) and the structure of nucleotide-free Ypt7 bound the Mon1/Ccz1 LD1 core (10) (PDB ID 5LDD).

Full details of methods are presented in *SI Appendix*, *Materials and Methods*.

## Supplementary Material

Appendix 01 (PDF)

## Data Availability

The cryo-EM density maps and models are deposited in the Electron Microscopy Data Bank www.emdatabank.org (EMD-16464, EMD-16463, EMD-16462, and EMD-16457) and the Protein Data Bank www.pdb.org (PDB ID 8C7G).
