# Supplementary material for: Structure of the metazoan Rab7 GEF complex Mon1–Ccz1–Bulli
Source: Proc Natl Acad Sci U S A. 2023 May 8;120(20):e2301908120. doi: 10.1073/pnas.2301908120 (PMC10193976; doi:10.1073/pnas.2301908120)
Supplement: Supplementary file 1 — Appendix 01 (PDF) [file pnas.2301908120.sapp.pdf]

**Supplementary Information**

**Methods**

*Protein expression and purification*

Recombinant MCBulli was produced using the biGBac expression system (1). Briefly, *Dm*Mon1-ΔN (residues 101-528) with an N-terminal GST-tag and PreScission cleavage site, *Dm*Ccz1-fl (residues 1-485) with an N-terminal 3xFlag-tag and *Dm*Bulli-fl (residues 1-642) with an N-terminal 6xHis-tag were first cloned into pLIB vectors. The expression cassettes were combined in pBIG1a and the resulting plasmid was transformed into *E. coli* DH10EMBacY. *Spodoptera frugiperda* 21 (*Sf*21) cells were transfected with bacmids to produce virus for expression.

*Sf*21 suspension cells were infected and grown in Spodopan medium (Pan Biotech) at 27°C for two to three days until 85% to 95% of the cells showed YFP fluorescence. Cells were harvested by centrifugation at 500 x*g* for 10 min, stored at -80°C and lyzed in PBS (10 mM $Na_2HPO_4$, 1.8 mM $KH_2PO_4$, 137 mM NaCl, 2.7 mM KCl) supplemented with 0.1% Nonidet P-40 (Applichem), 1mM Dithiothreitol and Protease Inhibitor Mix (Serva) using a LM20 microfluidizer (Microfluidics). The lysate was centrifuged at 40,000 x*g* for 30 minutes at 4°C and passed six times over glutathione agarose resin (Serva) equilibrated with PBS. After washing with PBS, the complex was cleaved off by incubation with PreScission protease overnight. To remove excess MC1 dimer, the elution fraction was passed four times over Ni-NTA agarose resin (Serva) equilibrated with PBS containing 10 mM imidazole. After washing with PBS, the remaining trimeric complex was eluted with PBS containing 300 mM imidazole. The elution fraction was concentrated to a final volume of 100 µL and loaded onto a Superdex 200i 5/150 GL size exclusion column (GE Healthcare) equilibrated with PBS (Fig. S1A). Peak fractions were analyzed by SDS-PAGE (Fig. S1B) and directly used for cryo-EM sample preparation.

*Cryo-EM sample preparation and data acquisition*

Sample quality was inspected by negative-stain electron microscopy as previously described (2). Micrographs were recorded manually, on a JEM2100plus transmission electron microscope (Jeol), operating at 200 kV and equipped with a Xarosa Emsis CMOS camera at a nominal magnification of 30,000, corresponding to a pixel size of 3.12 Å per pixel.

For cryo-EM, samples were concentrated to 0.75 - 1.0 mg/ml MCBulli and 3 µl were pipetted on freshly glow-discharged C-flat grids (Protochips; CF-1.2/1.3-3 Cu-50) and plunge frozen in liquid ethane using a Vitrobot Mark IV (Thermo Fisher) at 100 % relative humidity and 4°C.

The MCB-data was collected using a Glacios microscope (Thermo Fisher), operating at 200 kV and equipped with a Selectris energy filter (Thermo Fisher) with a slit with of 20 eV. Movies were recorded with a Falcon Mark IV direct electron detector (Thermo Fisher) at a nominal magnification of 130,000 corresponding to a calibrated pixel size

of 0.924 Å , and the data was saved in the electron-event representation (EER) format. The dose rate was set to 5.22 $e^-$ per pixel per second and a total dose of 50 $e^-$ per $Å^2$ was fractionated over 40 frames. 5,931 movies were collected automatically in EPU (Thermo Fisher) with a defocus range of -0.8 to -2.0 µm.

*Cryo-EM image processing*

The MCBulli dataset was processed in cryoSPARC (v3.2) (3). The processing workflow is presented in Fig. S2. Movies were preprocessed with patch-based motion correction, patch-based CTF estimation and filtered by the CTF fit estimates using a cutoff at 5 Å. A remaining stack of 4,842 micrographs was used for an initial round of Blob Picking. Well-defined 2D classes were selected and used for subsequent rounds of template-based 2D classification. 895K particles were extracted in a box of 320 pixels, and Fourier cropped to 160 pixels. Initial 3D reconstructions were generated by using different subsets of 2D classes.

Heterogeneous refinement (HR) was performed using the full set of 895K particles with 6 initial reconstructions as map inputs. Three well-defined reconstructions were re-extracted and re-centered using alignment shifts in a 320 px box. A consensus refinement for all 568K particles of 3.3 Å was obtained using non-uniform refinement (NUR) including per-particle defocus, global refinement of beam tilt and trefoil aberrations. A consecutive round of HR and NUR gave a final consensus refinement with a resolution of 3.2 Å from 390K particles.

To further improve the local resolution within the β-propeller of MCBulli, local refinements of the consensus map was performed using two different masks around the core of MCBulli and the β-propeller. To aid model building, a final composite map was generated with *phenix.combine_focused_maps*. All maps were subjected to B-factor sharpening within cryoSPARC alongside DeepEMhancer (4). Reported B-factors resulted from un-supervised auto-sharpening during refinement in cryoSPARC. Conformational flexibility was investigated trough the 3D-Variability Analysis tools (3D-VA) in cryoSPARC (v.3.2) (3). The final consensus map was used as input for the 3D-VA, including four variability components at a filtered resolution of 3.2 Å. Variability component one indicated a rocking motion around the Bulli-β-propeller region. To further separate stable particle clusters, mode one was separated into 10 clusters and individually reconstructed trough the 3D-VA Display job type. Stable clusters with well-aligning structural features were combined and polished by the non-uniform refinement procedure. No resolution increase was obtained following this processing route.

*Model building*

An initial model for MCBulli was generated using the multimer implementation of AlphaFold2 (5), placed in the composite map and fitted as rigid bodies by UCSF Chimera (6). The structure was manually edited in COOT (7) and iteratively refined using *phenix.real_space_refine* within Phenix (8). The cryo-EM density maps and models were deposited in the Electron Microscopy Data Bank (consensus map EMD-

16464, local refinement MCBulli core EMD-16463, local refinement MCBulli β-propeller EMD-16462, and the composite map EMD-16457) and the Protein Data Bank (PDB ID 8C7G). Data collection, image processing and model refinement statistics are summarized in Table S1.

*Pull-down assay*

Bulli mutants Q535A/D539R and Q535W/D539R were generated with Q5 site directed mutagenesis kit (NEB). Wild-type and mutant Bulli were cloned into a pcDNA3 vector with an N-terminal 3xFlag-GFP tag for expression in HEK293T cells. One 9 cm plate transfected with 6 µg plasmid of the respective constructs was lyzed with 800 µl PBS (10 mM $Na_2HPO_4$, 1.8 mM $KH_2PO_4$, 137 mM NaCl, 2.7 mM KCl) supplemented with 0.1% Nonidet P-40 (Applichem), 1 mM Dithiothreitol and Protease Inhibitor Mix (Serva) for 15 min at 4 °C. GST-*Dm*Mon1-ΔN (residues 101-528) and 3xFlag-*Dm*Ccz1-fl (residues 1-485) were expressed and purified essentially as described above for MCBulli, but directly used for pull-downs after immobilization on GSH beads and washing. 50 µl of GST-MC1 slurry (1:1) was added to cleared HEK293T cell lysates, incubated for 1 h at 4 °C. GSH-MC1 resin was pelleted (500 x*g*, 5 min, 4 °C) and washed three times with 500 µl PBS (10 mM $Na_2HPO_4$, 1.8 mM $KH_2PO_4$, 137 mM NaCl, 2.7 mM KCl). Samples were analyzed by SDS-PAGE and western blotting on PVDF membranes using a semi-dry system. Membranes were blocked with TBS (20 mM Tris, 150 mM NaCl, pH 7.6) supplemented with 5% milk, washed with TBS-T (20 mM Tris, 150 mM NaCl, 0.05% Tween20, pH 7.6) and detection was performed with α-GFP antibody (Proteintech Group, Inc, #66002) and α-mouse-HRP conjugate (Dako, #P0260) using ECL (Thermo Scientific, SuperSignal West Pico PLUS) on an Intas ChemoStar imager. Membranes were stripped with stripping buffer (0.2 M glycine, pH 2.2, 0.1% SDS, 1% Tween20) by heating for 30 sec and incubation at RT for 60 min and then stained with Coomassie.

*Computational modelling*

To generate a composite model of the pentameric *Drosophila* Rab5·GTP/Mon1/Ccz1/Bulli/Rab7 complex, we generated models of the dimeric Rab5/Mon1 and the trimeric Mon1/Ccz1/Rab7 complexes using AlphaFold multimer (5, 9) (Fig. S5). The GTPases were positioned on MCBulli by superposing the models with the experimental structure. The GTP pocket Rab5 was completed by copying the nucleotide from a superposition with human Rab5A (10) (PDB ID 1TU3). Switch I of Rab7 was manually rebuilt to adopt the conformation observed for nucleotide-free Ypt7 bound the Mon1/Ccz1 LD1 core (11) (PDB ID 5LDD).

## References


1. F. Weissmann, *et al.*, BiGBac enables rapid gene assembly for the expression of large multisubunit protein complexes. *Proc Natl Acad Sci U S A* **113**, E2564–E2569 (2016).
2. D. Januliene, A. Moeller, “Single-Particle Cryo-EM of Membrane Proteins” in (Humana, New York, NY, 2021), pp. 153–178.
3. A. Punjani, D. J. Fleet, 3D variability analysis: Resolving continuous flexibility and discrete heterogeneity from single particle cryo-EM. *J Struct Biol* **213**, 107702 (2021).
4. R. Sanchez-Garcia, *et al.*, DeepEMhancer: a deep learning solution for cryo-EM volume post-processing. *Commun Biol* **4**, 874 (2021).
5. J. Jumper, *et al.*, Highly accurate protein structure prediction with AlphaFold. *Nature* **596**, 583–589 (2021).
6. E. F. Pettersen, *et al.*, UCSF ChimeraX : Structure visualization for researchers, educators, and developers. *Protein Science* **30**, 70–82 (2021).
7. P. Emsley, B. Lohkamp, W. G. Scott, K. Cowtan, Features and development of Coot. *Acta Crystallogr D Biol Crystallogr* **66**, 486–501 (2010).
8. P. D. Adams, *et al.*, PHENIX: a comprehensive Python-based system for macromolecular structure solution. *Acta Crystallogr D Biol Crystallogr* **66**, 213–221 (2010).
9. R. Evans, *et al.*, Protein complex prediction with AlphaFold-Multimer. *bioRxiv*, 2021.10.04.463034 (2022).
10. G. Zhu, *et al.*, Structural basis of Rab5-Rabaptin5 interaction in endocytosis. *Nat Struct Mol Biol* **11**, 975–983 (2004).
11. S. Kiontke, *et al.*, Architecture and mechanism of the late endosomal Rab7-like Ypt7 guanine nucleotide exchange factor complex Mon1-Ccz1. *Nat Commun* **8**, 14034 (2017).

**Table S1**. EM data collection and refinement statistics.

| | **Consensus Map** | **MCBulli core** | **MCBulli β-propeller** | **Composite Map** |
|---|---|---|---|---|
| **Data Collection** | | | | |
| Accession number | EMD-16464 | EMD-16463 | EMD-16462 | EMD-16457 |
| Magnification | 130,000 | 130,000 | 130,000 | 130,000 |
| Voltage / kV | 200 | 200 | 200 | 200 |
| Dose / $e^{-}$ $Å^{-2}$ | 50 | 50 | 50 | 50 |
| Pixel size / Å | 0.924 | 0.924 | 0.924 | 0.924 |
| Defocus range / µm | -2.0 to -0.8 | -2.0 to -0.8 | -2.0 to -0.8 | -2.0 to -0.8 |
| Recorded movies | 5,931 | 5,931 | 5,931 | 5,931 |
| Initial particle images | 390,520 | 390,520 | 390,520 | 390,520 |
| Microscope | FEI Glacios | FEI Glacios | FEI Glacios | FEI Glacios |
| Camera | Falcon 4 | Falcon 4 | Falcon 4 | Falcon 4 |
| Energy Filter | Selectris | Selectris | Selectris | Selectris |
| **Image Processing** | | | | |
| Initial model | AlphaFold 2 | AlphaFold 2 | AlphaFold 2 | AlphaFold 2 |
| Processing software | cryoSPARC (v3.2) | cryoSPARC (v3.2) | cryoSPARC (v3.2) | cryoSPARC (v3.2) |
| Symmetry imposed | C1 | C1 | C1 | C1 |
| Resolution (FSC0.143) / Å | 3.2 | 3.2 | 3.7 | |
| Applied B-factor / $Å^2$ | -129 | -119 | -144 | |
| **Model Refinement** | | | | |
| PDB accession | | | | 8C7G |
| FSCmap-to-model(0.5)/Å | | | | 3.4 |
| MolProbity score | | | | 1.39 |
| Clash Score | | | | 3.99 |
| Atoms | | | | 11567 |
| Protein residues | | | | 1438 |
| Bonds (R.M.S.D.) | | | | |
| Length (Å) | | | | 0.004 |
| Angles (°) | | | | 0.586 |
| B-factors (min/max/mean) | | | | 22.67/107.34/46.89 |
| Ramachandran plot (%) | | | | |
| Favored | | | | 96.69 |
| Allowed | | | | 3.31 |
| Outliers | | | | 0 |
| Rotamer outliers (%) | | | | 0 |

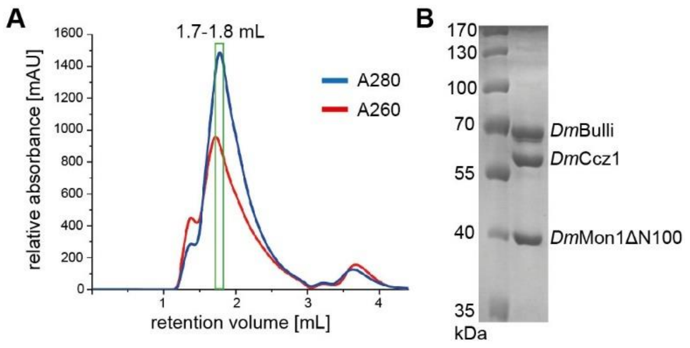


**Figure S1**: Purification of *Drosophila melanogaster* Mon1/Ccz1/Bulli. (**A**) Chromatogram from size exclusion of MCBulli using a Superdex 200i 5/150 GL column (GE Healthcare) equilibrated with PBS. (**B**) SDS-PAGE analysis of purified MCBulli used for cryo-EM analysis.

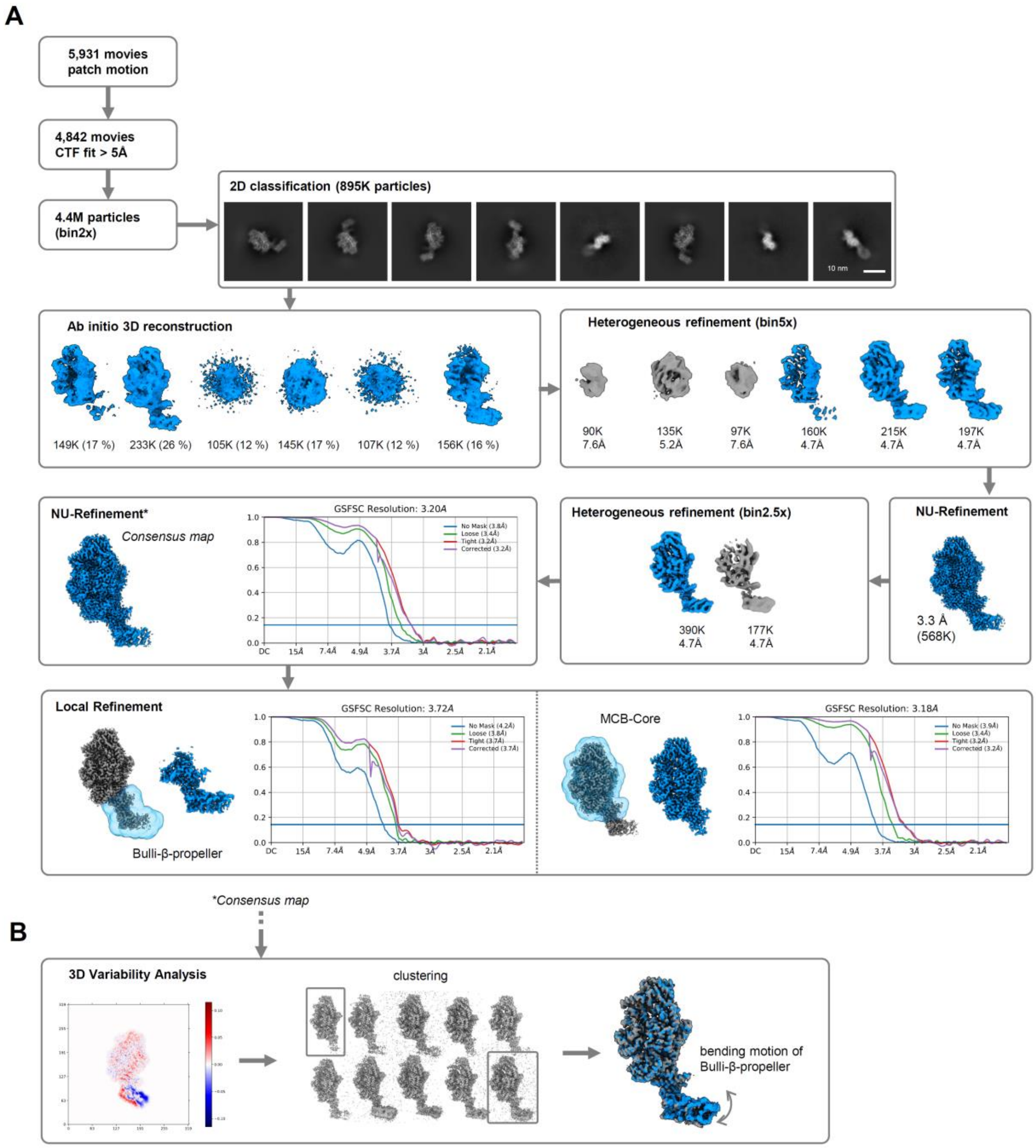


**Figure S2**: Cryo-EM of *Drosophila melanogaster* Mon1/Ccz1/Bulli. (**A**) Cryo-EM data processing workflow with representative 2D class averages of MCBulli. Resolution-estimation based on FSC =0.134 criterion. (**B**) Sub-classification strategy from the consensus refinement using cryoSPARC 3D variability analysis. All processing was performed using cryoSPARC v3.2.

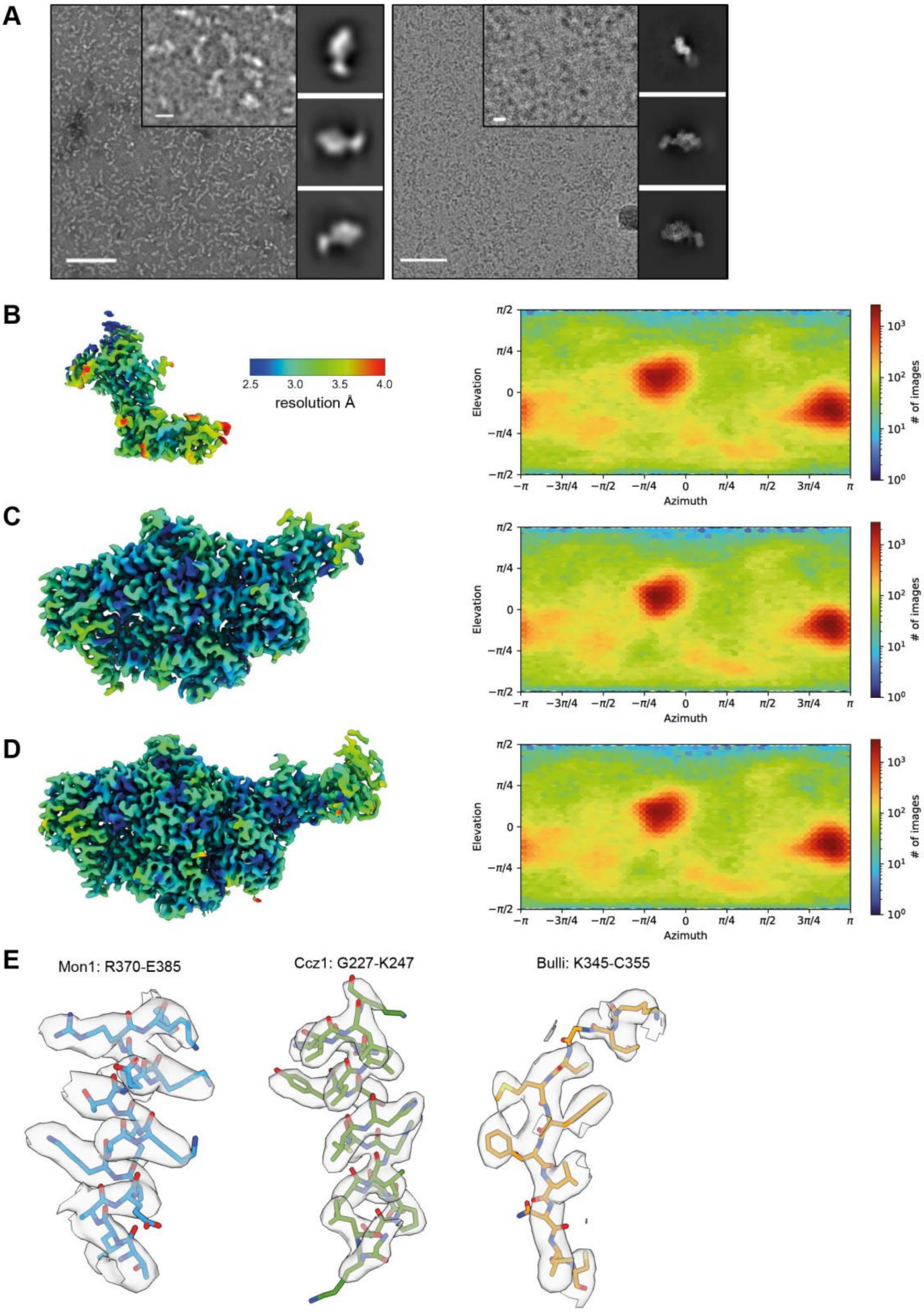


**Figure S3**: Cryo-EM data validation. (**A**) Representative micrographs from negative-stain (left) and cryo-EM (right) with 2D class averages. Scale bars are 100 nm in the micrographs and 10 nm in the magnified view. Local resolution estimation and heatmap of the orientation distribution of (**B**) the local density map around Bulli beta-propeller (**C**) the local density map of the MCB-core and (**D**) the consensus refinement. (**E**) Model/map fit of selected areas within each subunit. Used densities correspond to the local refinements from B for Bulli and C for Mon1 and Ccz1.

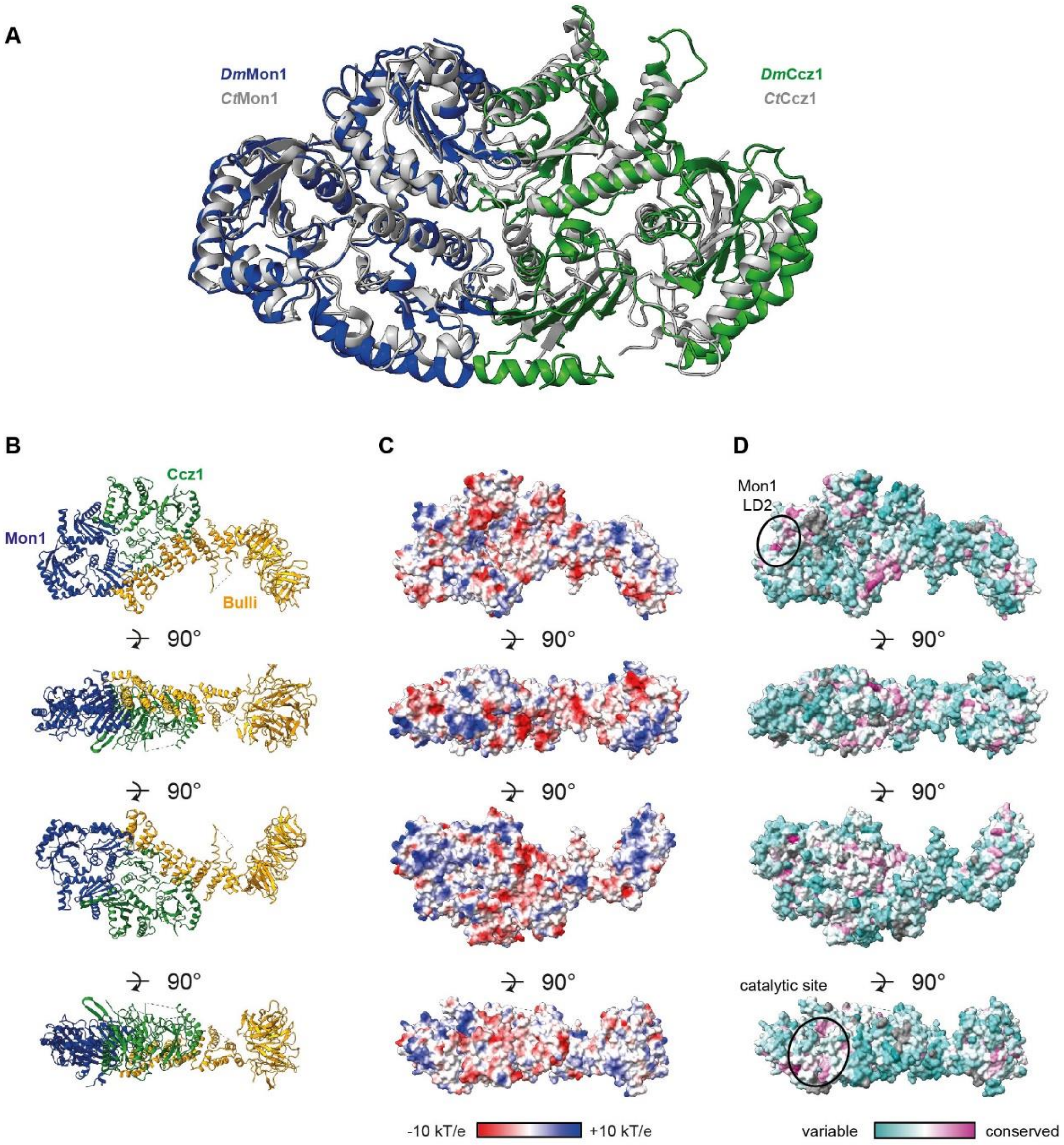


**Figure S4**: (**A**) Superposition of the TLD core subcomplex of *C. thermophilum* and *D. melanogaster*. (**B**) Four orthogonal views of the model of the complex, (**C**) the Coulomb surface potential and (**D**) surface conservation shown at identical orientations. Prominent patches are labeled.

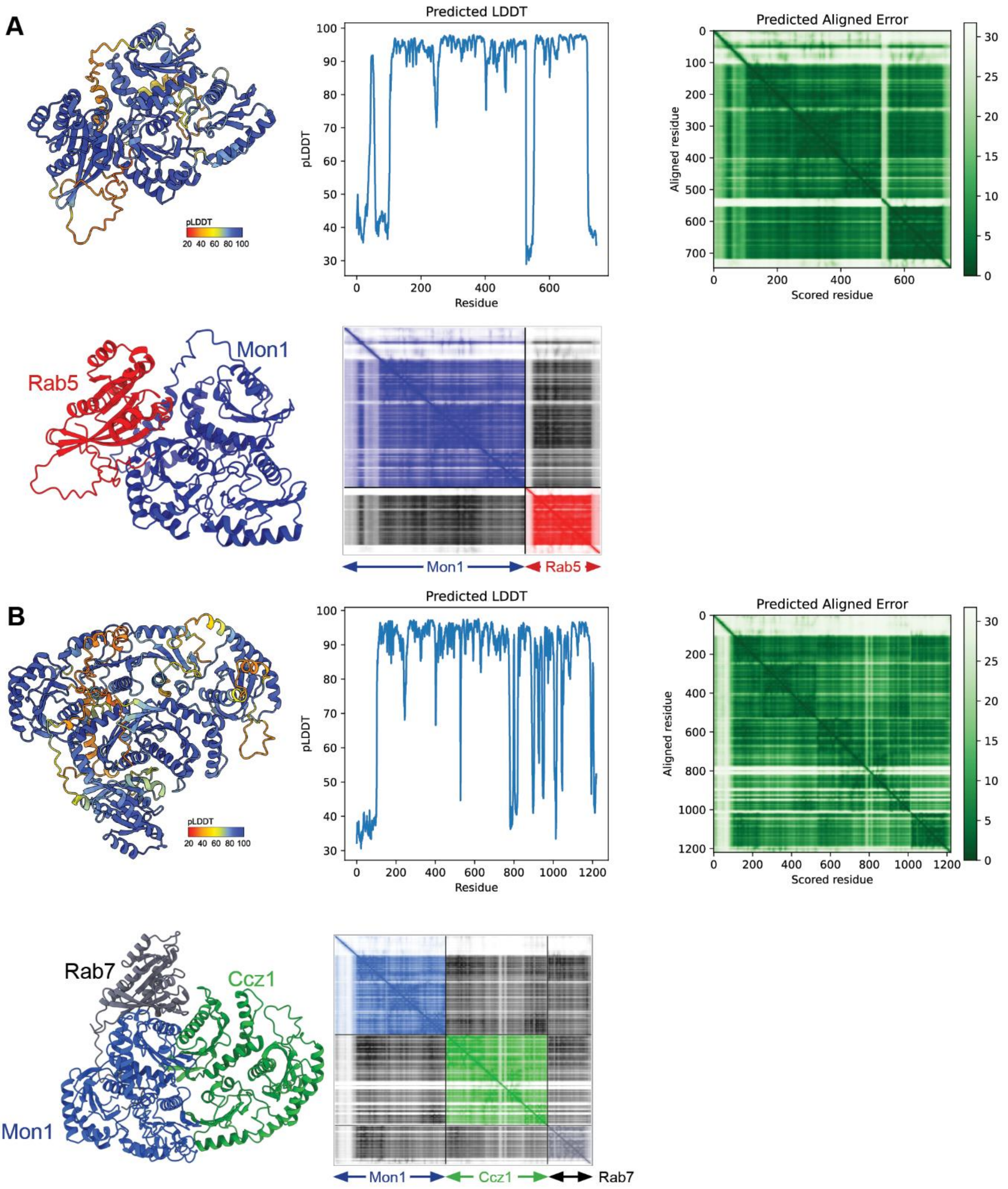


**Figure S5**: Validation of the AF2-predicted dmM5 and dmMC7 complex structures. Predicted model of dmM5 (A) and dmMC7 (B) represented as cartoons, color-coded according to the pLDDT values (per-residue local confidence on a scale from 0 – 100, top row) and colored by chain (bottom row). The inter-domain model accuracy is represented by the predicted aligned error (PAE) plot (expected position error 0 – 32 Å, top row) or colored by chain (bottom row).
